# Development and validation of a predictive model for acute myelitis secondary to hyperextension-induced spinal cord injury in pediatric patients

**DOI:** 10.3389/fneur.2025.1629920

**Published:** 2025-10-24

**Authors:** Honghui Lei, Haoran Yin, Fangyong Wang, Yang Yu, Wenjie Zhang, Meiling Cheng, Sitong Su

**Affiliations:** ^1^Department of Spine Surgery, Beijing Bo’ai Hospital, China Rehabilitation Research Center, Beijing, China; ^2^School of Rehabilitation, Capital Medical University, Beijing, China; ^3^Rehabilitation Medicine Center, The Second Affiliated Hospital and Yuying Children’s Hospital, Wenzhou Medical University, Wenzhou, China; ^4^University of Health and Rehabilitation Sciences, Qingdao, Shandong, China; ^5^School of Biological Science and Medical Engineering, Beihang University, Beijing, China; ^6^Department of Orthopaedics, Qilu Hospital, Cheeloo College of Medicine, Shandong University, Jinan, Shandong, China; ^7^School of Rehabilitation Medicine, Shandong University of Traditional Chinese Medicine, Jinan, China

**Keywords:** pediatric acute hyperextension spinal cord injury, pediatric myelitis, children, identifying disease, improving prognosis, predictive factors

## Abstract

**Background:**

The incidence of pediatric acute hyperextension-induced spinal cord injury (PAHSCI) is increasing in China, with some cases complicated by acute transverse myelitis (ATM). As predictive tools are lacking, this study aims to develop a clinical-imaging nomogram to assess ATM risk and support precision diagnosis and treatment in PAHSCI.

**Methods:**

We retrospectively analyzed clinical data from patients under 14 years of age diagnosed with thoracic PAHSCI between January 2012 and January 2023. All patients underwent lumbar puncture, gadolinium-enhanced imaging, and whole-spine MRI. Clinical history and imaging findings were collected, and the diagnosis of ATM was determined according to the Transverse Myelitis Consortium Working Group criteria. Patients were randomly assigned to training and validation cohorts in a 7:3 ratio. Least absolute shrinkage and selection operator (LASSO) regression was used to identify potential risk factors for ATM, which were then incorporated into a multivariable logistic regression model to construct a predictive nomogram. Model discrimination and calibration were assessed using the area under the receiver operating characteristic curve (AUC), calibration plots, and Brier scores. Internal validation was performed via 1,000-bootstrap resampling to generate 95% confidence intervals. Model goodness-of-fit was evaluated with the Hosmer–Lemeshow test, and clinical utility was assessed using decision curve analysis (DCA).

**Results:**

LASSO regression and multivariate logistic regression identified five predictors: age, fall, latent activity, flow void, and pinprick sensation score, which were used to construct a nomogram for estimating the risk of ATM in PAHSCI patients. The model demonstrated strong discriminative performance, with AUCs of 0.876 (95% CI: 0.803–0.950) in the training set and 0.844 (95% CI: 0.709–0.979) in the validation set. Calibration was satisfactory in both cohorts, as evidenced by the Hosmer–Lemeshow test (training: *χ*^2^ = 5.638, *p* = 0.776; validation: *χ*^2^ = 9.666, *p* = 0.378) and low Brier scores (0.138 and 0.167, respectively). Decision curve analysis indicated substantial net clinical benefit within risk thresholds of 8%–99% in the training cohort and 6%–71% in the validation cohort.

**Conclusion:**

We developed a preliminary nomogram demonstrating strong predictive accuracy for estimating ATM risk in PAHSCI patients, thereby enabling clinicians to adopt individualized therapeutic strategies.

## 1 Introduction

Pediatric acute hyperextension-induced spinal cord injury (PAHSCI) refers to a subtype of spinal cord injury without radiographic abnormalities (SCIWORA) occurring in children, typically following participation in activities such as dance, surfing, or taekwondo. It predominantly affects children under the age of 14 and is characterized by spinal cord damage at the thoracic level or below, without overt external trauma (1, 2). In China, the incidence of PAHSCI has been rising in recent years and now accounts for over 50% of all pediatric SCIWORA cases (3–5). Clinically, PAHSCI often presents with limb paralysis, sensory disturbances, and sphincter dysfunction (6). Magnetic resonance imaging (MRI) has long been considered the gold standard for diagnosis, typically revealing spinal cord edema, hemorrhage, or atrophy (7). Recent studies have also identified imaging features suggestive of spinal venous hypertension in PAHSCI patients (8, 9), with typical T2-weighted imaging findings including intramedullary hyperintensity, perimedullary hypodensity, and prominent perimedullary flow voids.

A distinct subset of PAHSCI cases is also diagnosed with acute transverse myelitis (ATM). These patients typically exhibit sensory, motor, and autonomic dysfunction, similar to classic ATM presentations (10). The diagnosis of ATM is generally supported by a preceding viral prodrome—such as fever, upper respiratory infection, or diarrhea—or evidence of autoimmune response, along with abnormal spinal MRI findings, elevated cerebrospinal fluid (CSF) cell counts, or increased CSF immunoglobulin G index (11, 12). Gadolinium-enhanced abnormalities on spinal or cranial MRI may further assist in diagnosis (13). However, in clinical practice, particularly in resource-limited settings such as community hospitals, ATM is often diagnosed based primarily on MRI findings due to limited access to comprehensive testing. Given insufficient clinical awareness, there is a tendency to misclassify typical PAHSCI as ATM-type PAHSCI when relying solely on imaging, potentially leading to diagnostic inaccuracies (14).

To date, few studies have focused on differentiating classical PAHSCI from its ATM-associated subtype, and there remains a lack of reliable tools to predict the risk of ATM in patients with PAHSCI. Nomograms represent robust statistical models that integrate multiple risk factors to generate individualized risk estimates in a graphical format (15). Previous work has developed prognostic models for neurological recovery in pediatric PAHSCI based on multivariate logistic regression analyses (16). However, no validated model currently exists for predicting the likelihood of ATM in PAHSCI cases. This study aims to identify key clinical, historical, and radiographic features associated with ATM-type PAHSCI and to construct a predictive nomogram that may aid in the diagnosis, differentiation, and management of this condition.

## 2 Materials and methods

### 2.1 Ethics statement and general information

We conducted this study in compliance with the principles of the Declaration of Helsinki. The study’s protocol was reviewed and approved by the Institutional Review Board of the China Rehabilitation Research Center (IRB No. 2022-023-1). The STROBE guideline was used for the manuscript.

This retrospective study included children aged 14 years or younger who were diagnosed with thoracic PAHSCI and treated at the China Rehabilitation Research Center, Beijing, China, between January 2012 and January 2023. The inclusion criteria were as follows: (1) absence of neurological symptoms, including motor, sensory, or sphincter dysfunction, within 2 weeks prior to injury; (2) clinical signs and symptoms of spinal cord injury following sports-related trauma, consistent with the 2011 International Standards for Neurological Classification of Spinal Cord Injury (17); (3) absence of vertebral fracture or dislocation on radiographic or CT imaging. The exclusion criteria were: (1) incomplete clinical or imaging data; (2) spinal cord injury due to other causes, including space-occupying lesions, vascular malformations, surgical trauma, penetrating injuries, birth trauma, congenital spinal anomalies, or systemic diseases (e.g., leukemia, connective tissue disorders); (3) concomitant neurological disorders, such as acute disseminated encephalomyelitis or neuromyelitis optica. All patients underwent lumbar puncture, gadolinium-enhanced MRI, and brain MRI to exclude underlying conditions.

### 2.2 Methods

#### 2.2.1 Data collection

Clinical and imaging data were collected as potential predictors for the clinical prediction model. The clinical and pathological characteristics included in this study were: age, gender, length of admission, fall, latent period, latent paresthesia, latent activity, flow void, spinal cord atrophy, spinal atrophy segment length, long T2 signal in spinal cord, spinal edema segment length, lower limb muscle tone, lower limb tendon reflex, nerve injury plane, American Spinal Injury Association Impairment Scale (AIS) grade, pinprick sensation score, light touch score, sensory score, and muscle strength score of key lower limb muscles. Whole-spine MRI scans were performed using a 3.0 Tesla scanner. Axial images were assessed for flow voids, while sagittal images were evaluated for spinal cord edema and atrophy. Two investigators independently reviewed the medical records and imaging studies to confirm the diagnosis of PAHSCI.

#### 2.2.2 Diagnosis of ATM

The diagnosis of ATM was established based on the criteria defined by the Transverse Myelitis Consortium Working Group (18). The diagnostic criteria included: (1) a history of febrile illness, upper respiratory tract infection, diarrhea, or recent vaccination within the preceding weeks; (2) development of sensory, motor, or autonomic dysfunction attributable to spinal cord injury; (3) exclusion of extra-axial compressive causes by MRI or myelography; (4) spinal cord inflammation confirmed by CSF analysis, including pleocytosis, elevated immunoglobulin G index, or gadolinium enhancement on MRI.

### 2.3 Data analysis

The time difference between the first symptom and admission was calculated as the length of admission. The latent period was defined as the time between the onset of the first symptoms and paralysis of the lower limbs. Latent paresthesia was defined as numbness, pain in the lower limbs, or lower back pain during the latency period. Latent activity was defined as continued hyperextension exercises during this phase. The nerve injury planes were divided into upper thoracic vertebrae (T1–T6) and lower thoracic vertebrae (T7–T12). The 2011 International Standard for Neurological Classification of Spinal cord Injury was applied to the degree of sensorimotor impairment at admission and discharge, and AIS grade, acupuncture sensation, light touch, and muscle strength scores of key muscles of lower limbs were obtained (17). The sensory score was the sum of acupuncture and light touch. AIS grade A is complete injury, others are incomplete injury.

### 2.4 Statistical analysis

SPSS 25.0 software, with its intuitive interface and comprehensive statistical tools, was employed to perform data preprocessing, distribution testing, descriptive analysis, and standard hypothesis testing. The measurement data conform to normal distribution, expressed as ( $\bar{x}$ ± s); nonconformities were represented by M (P25, P75), and Mann–Whitney U test was used. The *Z* value was calculated using the normal approximation method, and the *p* value was reported. Categorical data were expressed as frequencies and percentages *N* (%) and analyzed using the chi-square (*χ*^2^) test. The *χ*^2^ and *p* values were reported. Statistical significance was defined as *p* ≤ 0.05.

R 4.3.2 software was used for advanced modeling and validation owing to its powerful open-source package ecosystem and high programming flexibility. Patients were randomly divided into a training cohort and a validation cohort at a 7:3 ratio. Least absolute shrinkage and selection operator (LASSO) regression was employed to identify meaningful combinations of risk factors predictive of ATM in PAHSCI patients. As a shrinkage estimation method, LASSO reduces dimensionality by applying a penalty function, which compresses regression coefficients and sets some to zero, thereby facilitating variable selection (19, 20). Clinical variables with nonzero coefficients in the LASSO model were selected as optimal predictors (21). This method balances model complexity and predictive accuracy, effectively preventing overfitting while mitigating multicollinearity and avoiding the subjective bias of stepwise regression and the variable redundancy of ridge regression. These predictors were then incorporated into a multivariate logistic regression model to construct a nomogram. Statistical significance was defined as *p* ≤ 0.05.

The discriminative performance of the nomogram was assessed in both the training and validation cohorts by plotting receiver operating characteristic curves and calculating the area under the curve (AUC). To evaluate the robustness of the AUC estimates, 1,000 bootstrap resampling were used to derive the 95% confidence intervals (CI). Calibration was assessed using calibration curves and Brier scores, both generated through 1,000 bootstrap replicates, to visualize the agreement between predicted probabilities and actual outcomes. The Hosme–Lemeshow goodness-of-fit test (HLGT) was applied to further evaluate model calibration. An AUC greater than 0.75 was considered indicative of good discriminative ability, while values between 0.5 and 0.75 were regarded as acceptable (22). A non-significant HLGT (*p* > 0.05), a calibration curve closely aligned with the diagonal, and a Brier score <0.25 were considered indicative of good model calibration.

Finally, the clinical utility of the nomogram was evaluated by calculating net benefits and constructing decision curve analyses (23, 24).

## 3 Results

**Figure 1:**
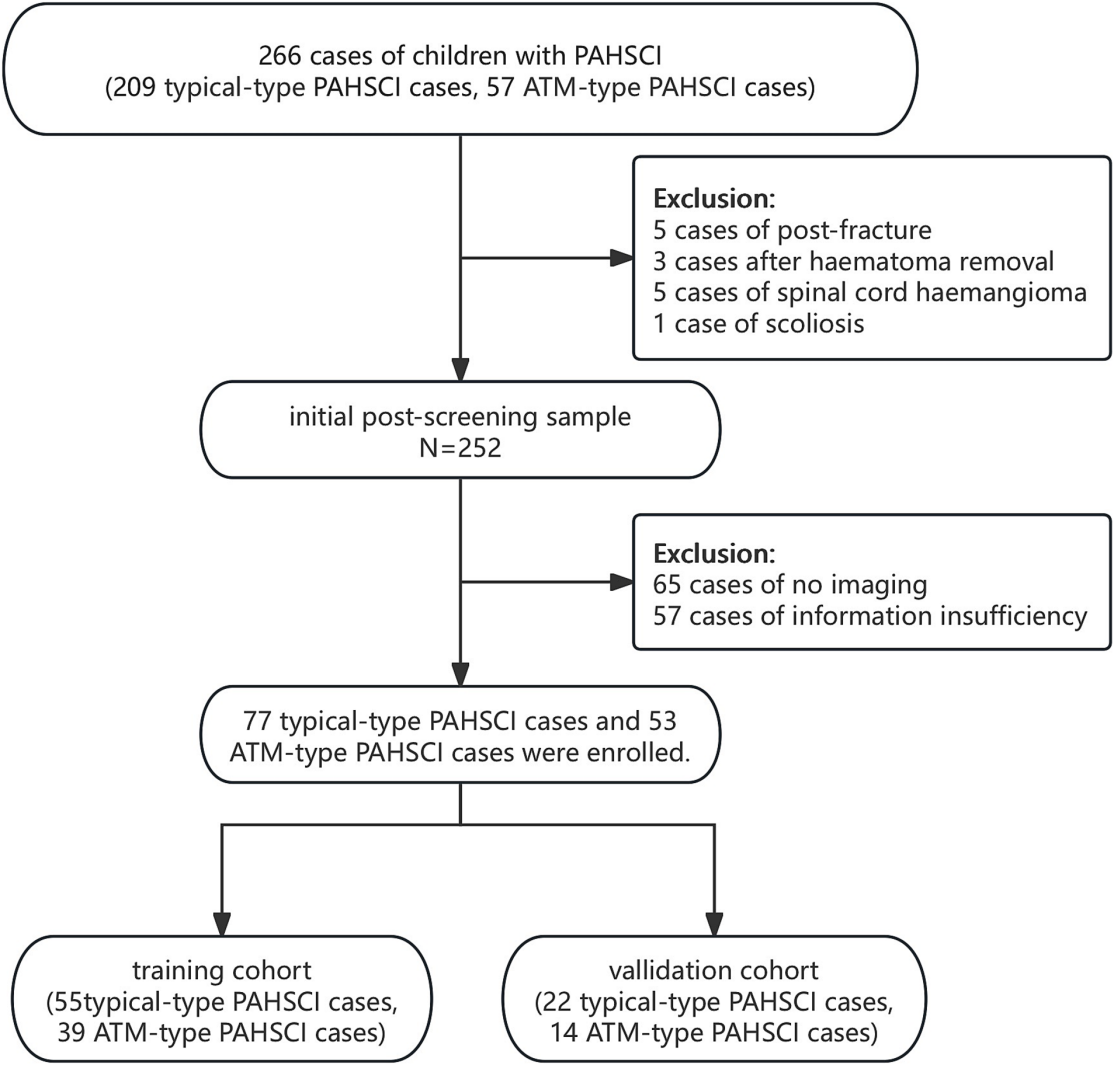
The enrollment flow chart of the study.

By browsing the medical records, 266 cases of children with PAHSCI or ATM under the age of 14 were obtained. Afterwards, we excluded cases including 65 cases of no imaging, five cases of post-fracture, three cases after haematoma removal, five cases of spinal cord haemangioma, one case of scoliosis and 57 cases of information insufficiency. Finally, 130 cases were included, of which 77 cases were from the typical-type PAHSCI group and 53 cases were from the ATM-type PAHSCI group (Figure 1).

### 3.1 Imaging analysis

**Figure 2:**
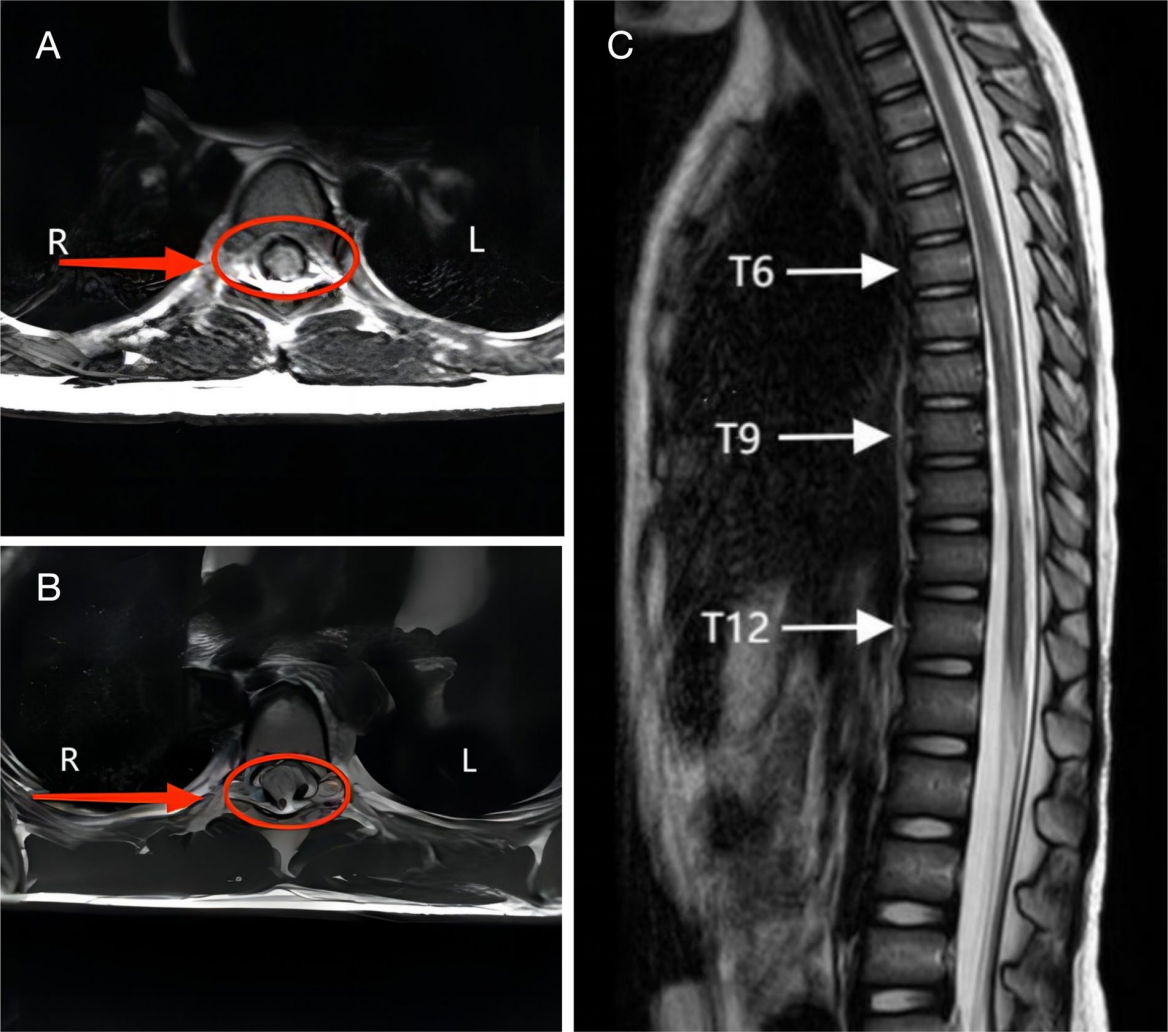
**(A)** A 5-year-old female developed lower limb pain and numbness 30 min after performing a backbending exercise, diagnosed with complete spinal cord injury. MRI T2WI of the T7 spinal cord showed the presence of the flow void with a coil sign around cord. **(B)** A 6-year-old female experienced immediate inability to move both lower limbs after a backbending exercise, diagnosed with incomplete spinal cord injury. MRI T2WI of the T10 spinal cord showed the presence of the flow void with a dot sign around cord. **(C)** A 5-year-old female showed immediate loss of movement in both lower limbs after a backbending exercise, diagnosed with complete spinal cord injury. MRI T2WI shows spinal cord edema in T6-12 and spinal cord atrophy in T9-12.

All patients underwent whole spine radiography, CT and MRI imaging. No vertebral fracture or subluxation was found on *X*-ray film or CT. MRI examination did not show any abnormalities in the morphology and signal of the vertebral body and intervertebral discs. In this study, the characteristics of spinal venous hypertension could be seen on some T2-weighted imaging images, intramedullary high-intensity signal and flow voids resembling dots or coils around cord (Figures 2A,B). In some cases, long T2 signals were seen in the spinal cord in sagittal sequences, suggesting spinal edema (Figure 2C). In addition, in some of the children, the spinal cord morphology was reduced in the sagittal sequence, suggesting spinal atrophy (Figure 2C).

### 3.2 Patient characteristics

Detailed clinical characteristics of the two patient groups are presented in Table 1. According to the Mann–Whitney U test and Pearson Chi-square test, several clinicopathological features—including age, length of admission, fall, latent period, latent activity, flow void, long T2 signal in spinal cord, and spinal edema segment length—were statistically significant (*p* < 0.05), suggesting a strong association with the occurrence of ATM in children with PAHSCI. A distinctive feature of the cohort was the marked female predominance (83%, 108/130), reflecting the primary injury mechanism in this population. Most injuries were related to dance training, an activity with high female participation in China. Accordingly, although the model demonstrated strong internal performance, its predictive accuracy may be most relevant to female patients with dance-associated PAHSCI. Its generalizability to male patients or to PAHSCI arising from other causes requires validation in more gender-balanced, multicentre cohorts.

**Table 1:** General conditions of children with typical-type PAHSCI (*n* = 77) and ATM-type PAHSCI (*n* = 53).

	Typical-type (*n* = 77)	ATM-type (*n* = 53)	χ^2^/Z	*p*-value
Age/y	7 (6, 8.5)	5 (4, 7)	−4.740	<0.001^a^
Gender/*n*				
Men (1)	10 (0.130)	12 (0.226)	2.081	0.149^b^
Women (0)	67 (0.870)	41 (0.774)		
Length of admission/d	24 (15, 53.5)	60 (32.5, 111)	−4.213	<0.001^a^
Fall/*n*				
Yes (1)	43 (0.558)	12 (0.226)	14.179	<0.001^b^
No (0)	34 (0.442)	41 (0.774)		
Latent period/h				
<0.5 (0)	51 (0.662)	20 (0.377)	10.286	0.001^b^
≥0.5 (1)	26 (0.338)	33 (0.623)		
Latent paresthesia/*n*				
Yes (1)	59 (0.766)	35 (0.660)	1.757	0.185^b^
No (0)	18 (0.234)	18 (0.340)		
Latent activity/*n*				
Yes (1)	36 (0.468)	8 (0.151)	14.052	<0.001^b^
No (0)	41 (0.532)	45 (0.849)		
Flow void/*n*				
Yes (1)	59 (0.766)	18 (0.340)	23.660	<0.001^b^
No (0)	18 (0.234)	35 (0.660)		
Spinal cord atrophy/*n*				
Yes (1)	24 (0.312)	24 (0.453)	2.685	0.101^b^
No (0)	53 (0.688)	29 (0.547)		
Spinal atrophy segment length	0 (0, 3)	0 (0, 3.5)	−1.427	0.154^a^
Long T2 signal in spinal cord/*n*				
Yes (1)	37 (0.481)	35 (0.660)	4.110	0.043^b^
No (0)	40 (0.519)	18 (0.340)		
Spinal edema segment length	0 (0, 6.5)	6 (0, 8)	−2.193	0.028^a^
Lower limb muscle tone/*n*				
Exist (1)	12 (0.156)	10 (0.189)	0.241	0.624^b^
Disappear (0)	65 (0.844)	43 (0.811)		
Lower limb tendon reflex/*n*				
Exist (1)	15 (0.195)	7 (0.132)	0.879	0.349^b^
Disappear (0)	62 (0.805)	46 (0.868)		
Nerve injury plane/*n*				
T1–T6 (1)	25 (0.325)	13 (0.245)	0.957	0.328^b^
T7–T12 (0)	52 (0.675)	40 (0.755)		
AIS grade/*n*				
Complete (1)	57 (0.740)	41 (0.774)	0.188	0.665^b^
Incomplete (0)	20 (0.260)	12 (0.226)		
Pinprick sensation score	66 (58.5, 72)	64 (60, 68)	−1.182	0.237^a^
Light touch score	68 (62, 74)	64 (60, 68.5)	−1.346	0.178^a^
Sensory score	134 (122, 145.5)	128 (120, 136)	−1.209	0.227^a^
Muscle strength score of key lower limb muscles	0 (0, 0)	0 (0, 0)	−0.004	0.997^a^

a Mann–Whitney U test.

b Pearson Chi-square test.

y, years; *n*, number of cases; d, days; h, hours.

### 3.3 Selection of risk factors

**Figure 3:**
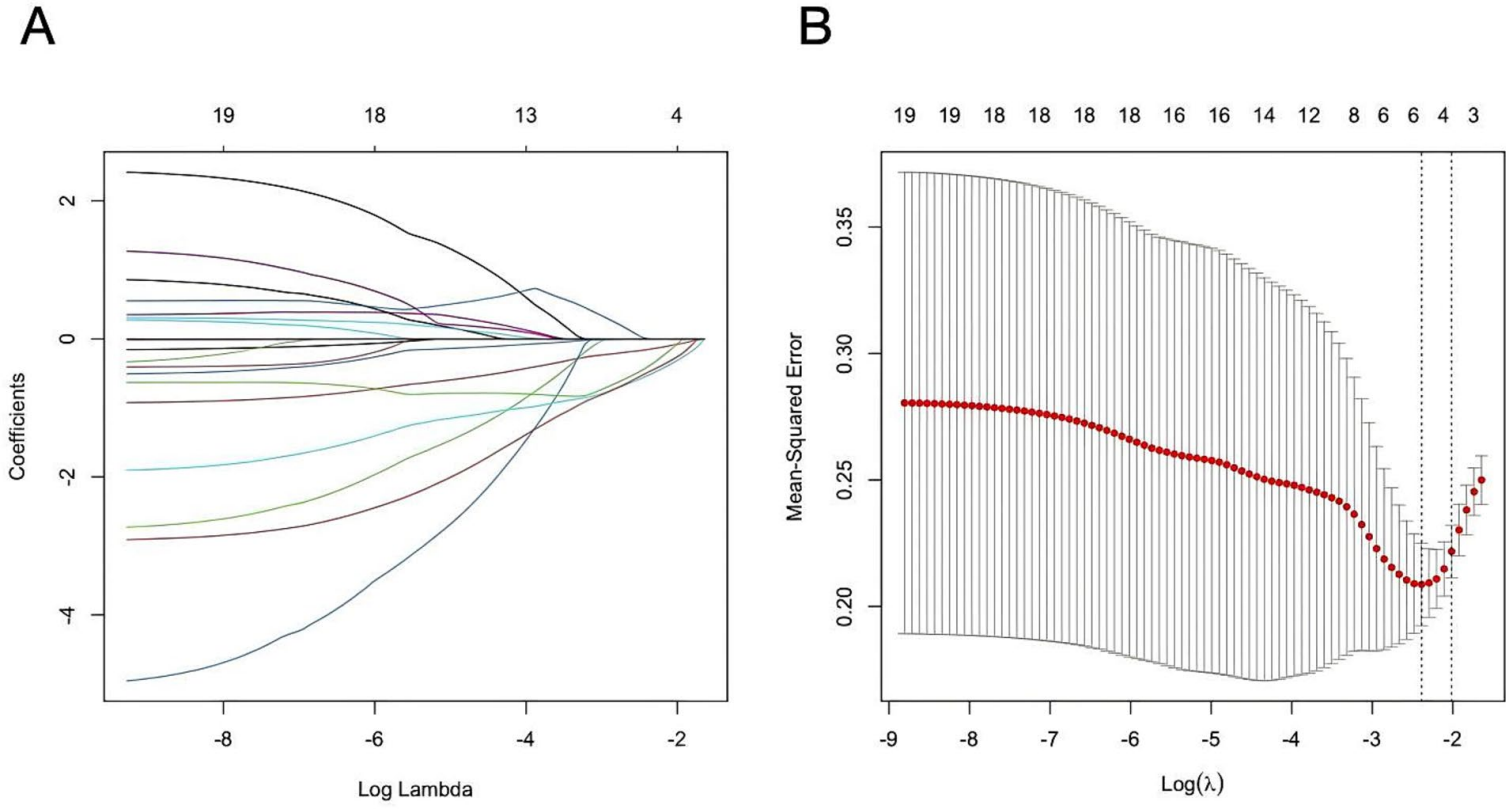
Procession of LASSO. **(A)** Regression coefficient plot. **(B)** Cross-validation plot.

Among the 20 clinicopathological variables evaluated, six were selected from the training cohort using the LASSO regression model, as they retained non-zero coefficients (Figures 3A,B). These variables—age, fall, latent activity, flow void, pinprick sensation score, and spinal cord atrophy—were identified as potential predictors (Supplementary Datasheet 1).

### 3.4 Construction of the nomogram

**Figure 4:**
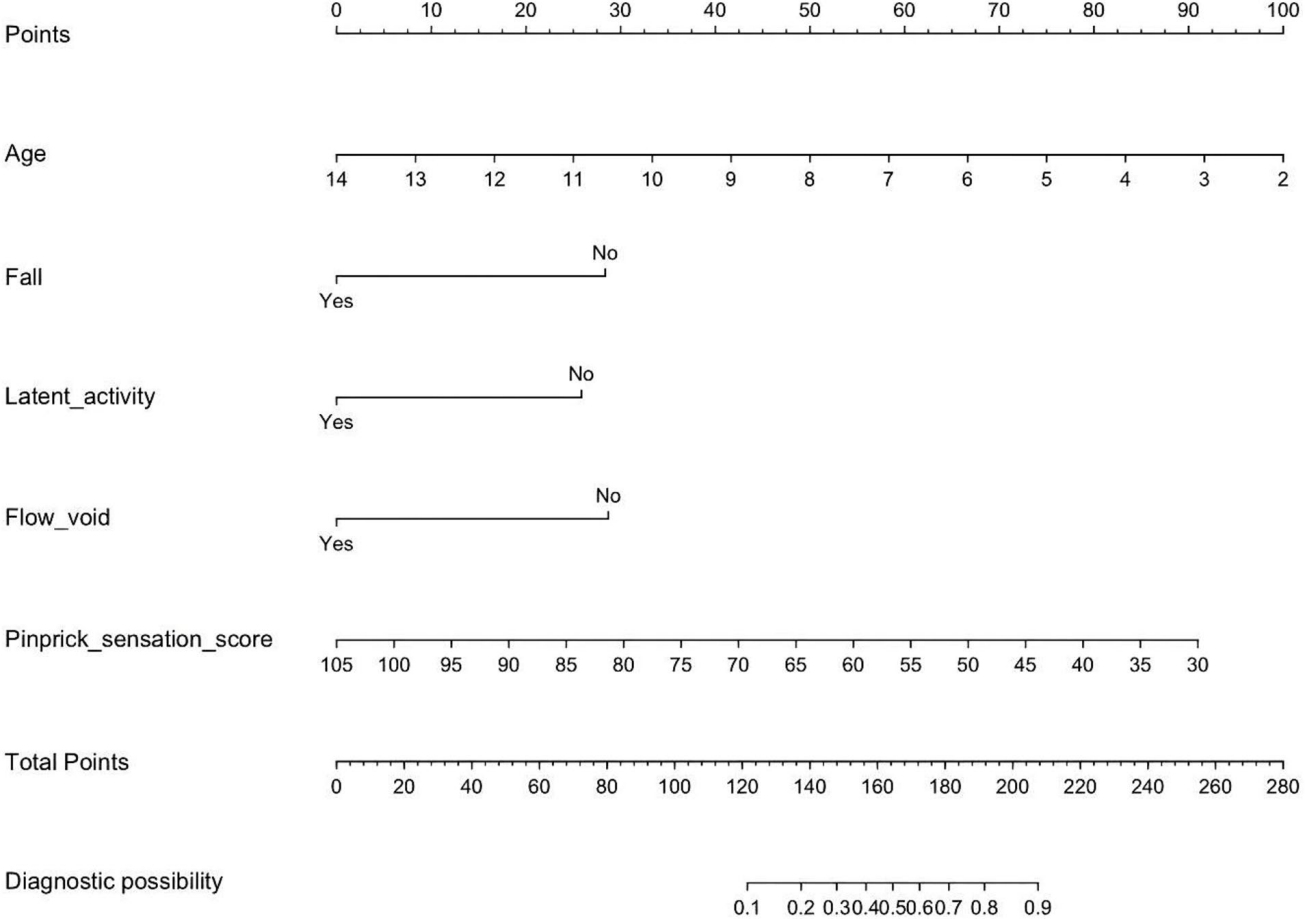
Nomogram for predicting myelitis in children after hyperextension exercise.

**Figure 5:**
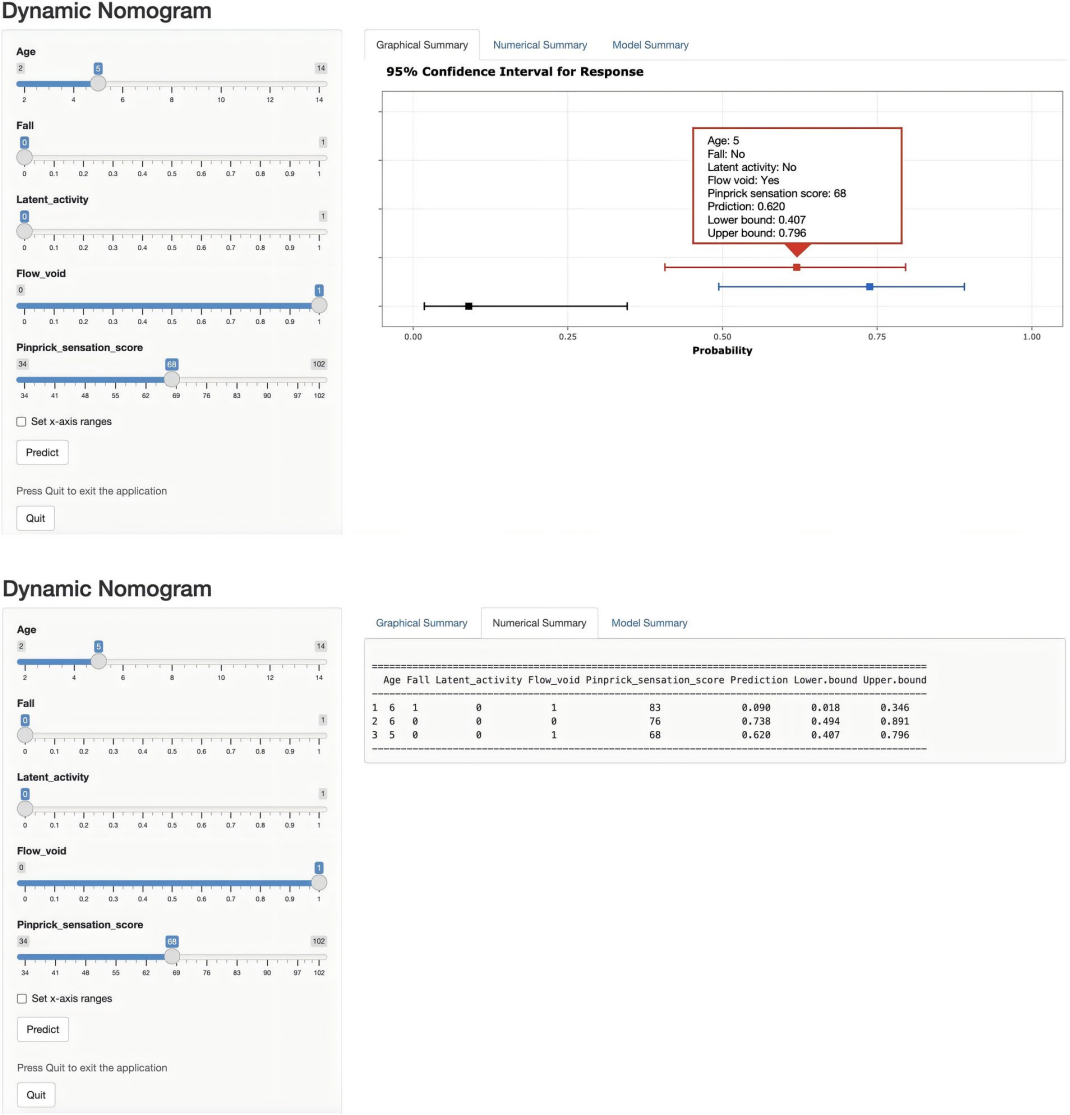
Dynamic nomogram for predicting the risk of ATM in children with PAHSCI. The plot depicts the estimated probability of ATM, with 95% confidence intervals, in patients with PAHSCI. Corresponding explanatory variables and predicted probabilities are provided in the “Numerical Summary” tab.

Univariate and multivariate logistic regression analyses were performed on the predictors identified by LASSO regression, as shown in Table 2. In the end, five statistically significant variables—age, fall, latent activity, flow voids, and pinprick sensation—were incorporated into the final predictive model, which was subsequently visualized as a nomogram (Figure 4). To overcome the limitations of traditional nomograms with higher-order interactions, we developed a Shiny-based dynamic nomogram (Figure 5) (25). It predicts the risk of ATM in children with PAHSCI with 95% confidence intervals, displaying results both graphically and numerically, and is freely available at https://leihonghui.shinyapps.io/DynNomapp/.

**Table 2:** Univariate (x) and multivariate (y) logistic analysis of factors influencing the differentiation between typical-type and ATM-type PAHSCI.

	B.x	SE.x	OR.x	CI.x	Z.x	P.x	B.y	SE.y	OR.y	CI.y	Z.y	P.y
Age	−0.439	0.128	0.64	0.5–0.83	−3.445	0.001	−0.426	0.148	0.65	0.49–0.87	−2.872	0.004
Fall	−1.76	0.483	0.17	0.07–0.44	−3.646	<0.001	−1.45	0.616	0.23	0.07–0.78	−2.356	0.018
Flow void	−1.327	0.452	0.27	0.11–0.64	−2.938	0.003	−1.467	0.6	0.23	0.07–0.75	−2.444	0.015
Latent activity	−1.702	0.497	0.18	0.07–0.48	−3.422	0.001	−1.322	0.623	0.27	0.08–0.9	−2.123	0.034
Pinprick sensation score	−0.042	0.02	0.96	0.92–1	−2.148	0.032	−0.062	0.029	0.94	0.89–0.99	−2.128	0.033
Spinal cord atrophy	0.772	0.43	2.16	0.93–5.03	1.793	0.073	NA	NA	NA	NA	NA	NA

### 3.5 Evaluation of the nomogram performance

**Figure 6:**
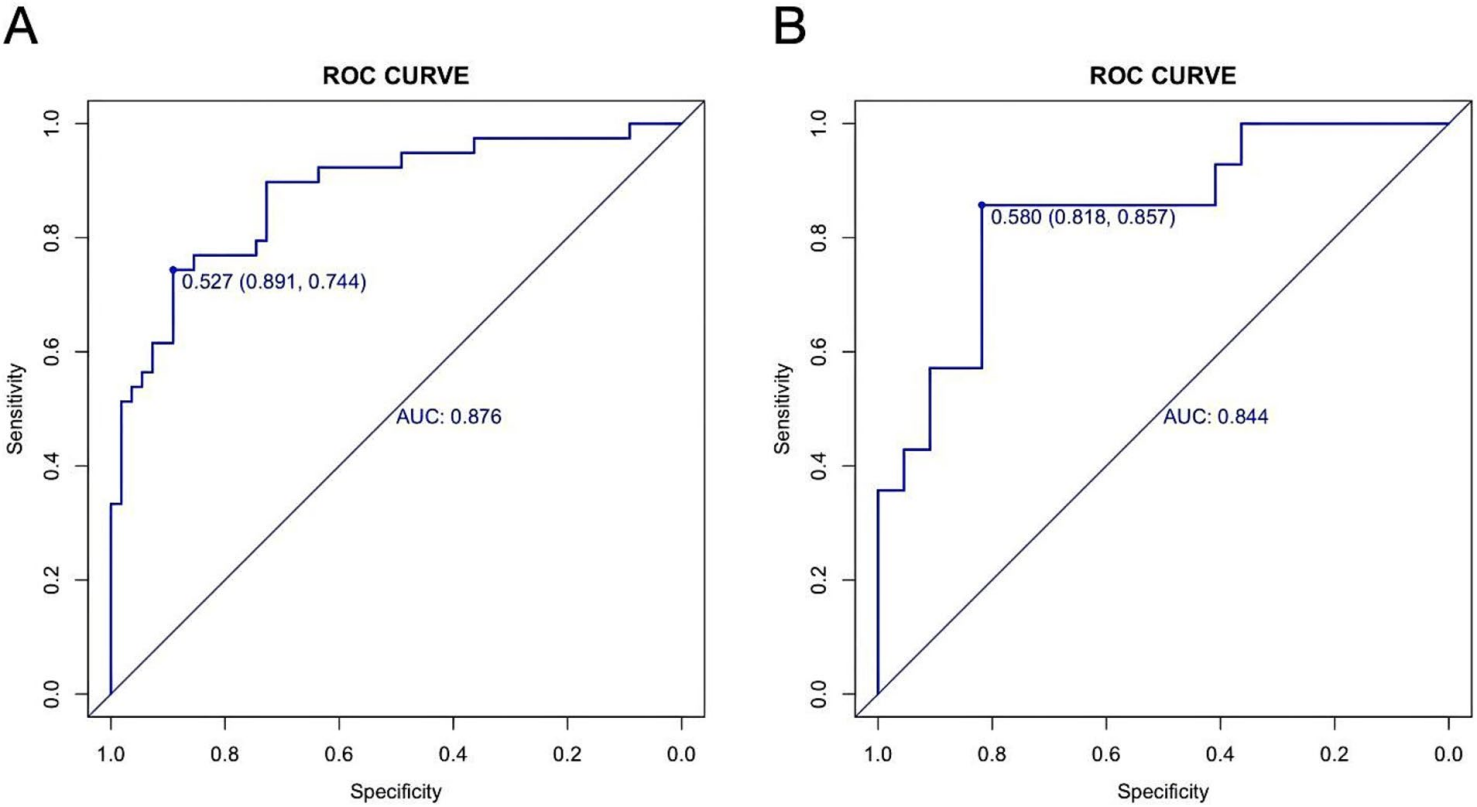
Receiver operating characteristic curves in the training **(A)** and validation **(B)** cohorts.

**Figure 7:**
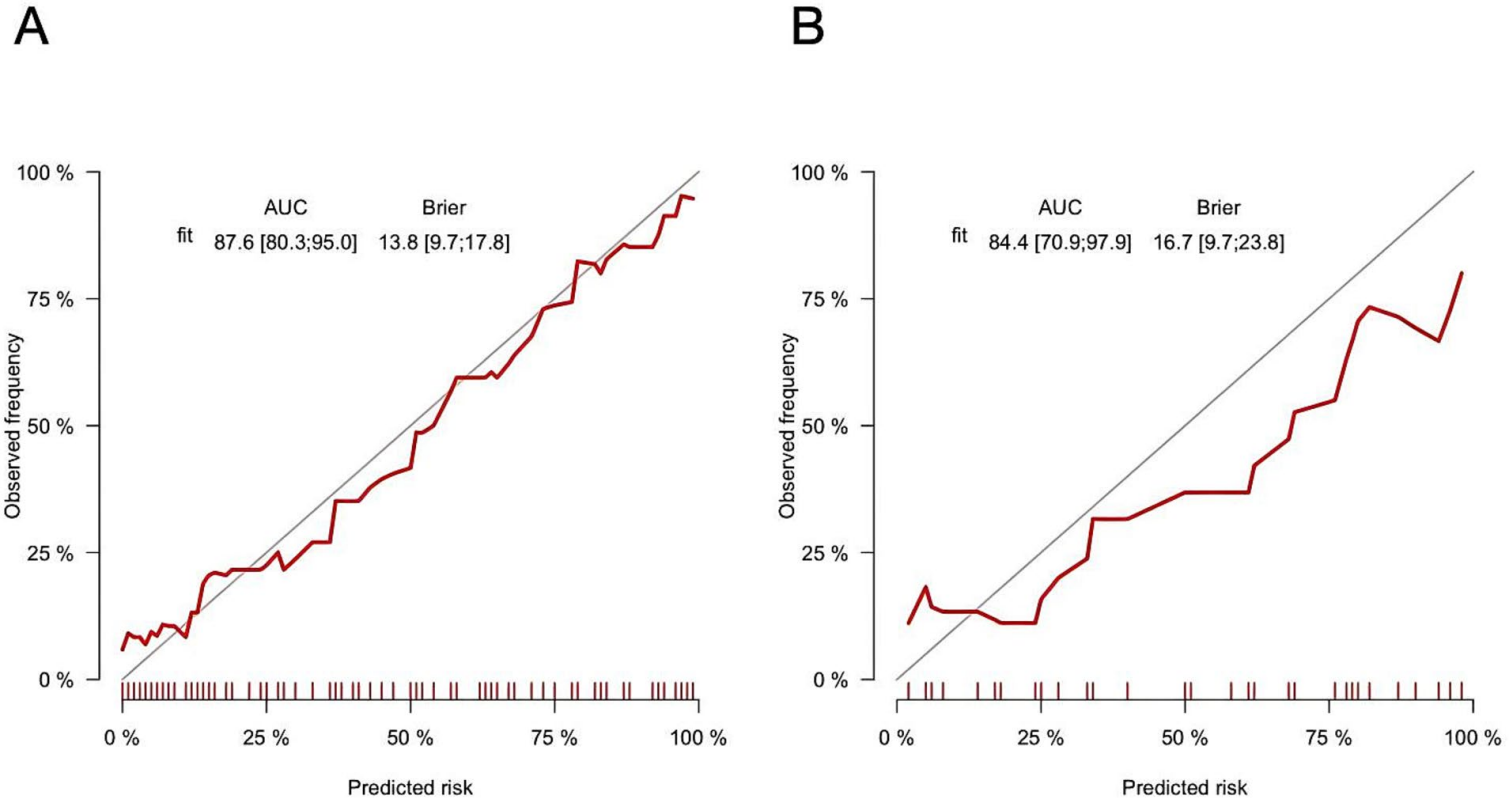
Training **(A)** and verification **(B)** cohorts of calibration curves for myelitis (bootstrap = 1,000).

The AUC was 0.876 (95% CI: 0.803–0.950) in the training cohort and 0.844 (95% CI: 0.709–0.979) in the validation cohort (Figures 6A,B), indicating strong discriminative ability. The calibration curves and HLGT results demonstrated strong agreement between predicted and observed risks of ATM in children with PAHSCI in both cohorts (Figures 7A,B). In the training cohort, the HLGT yielded *χ*^2^ = 5.638, *p* = 0.776, and Brier = 0.138; in the validation cohort, *χ*^2^ = 9.666, *p* = 0.378, and Brier = 0.167. These findings indicate that the nomogram exhibits excellent calibration and predictive performance in estimating the risk of ATM in PAHSCI patients.

### 3.6 Clinical utility

**Figure 8:**
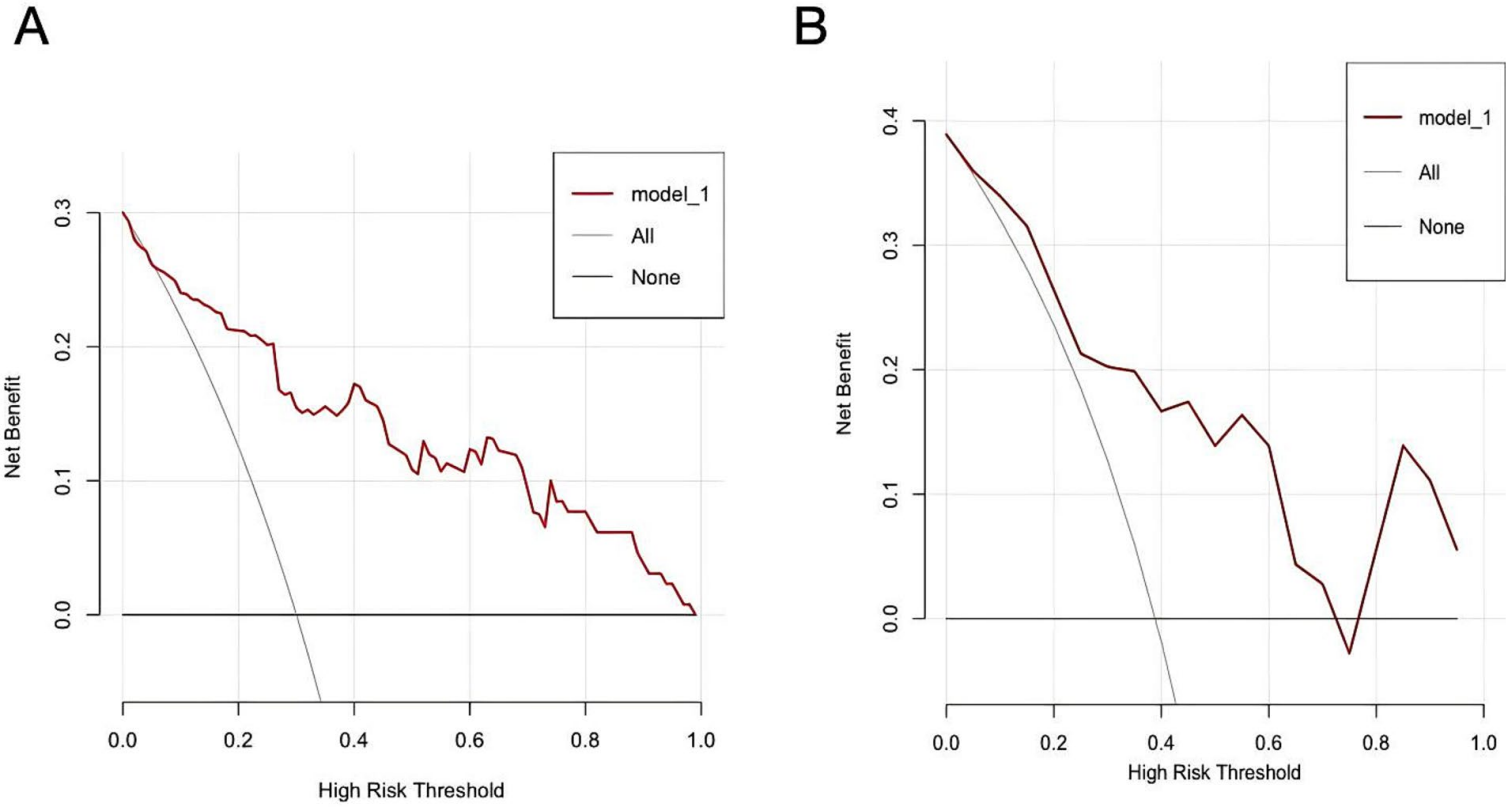
Decision curve analyses of myelitis in training **(A)** and validation **(B)** cohorts.

Decision curve analyses for both the training and validation cohorts is shown in Figures 8A,B. The nomogram provided a net clinical benefit in predicting the risk of ATM when the threshold probabilities ranged from 8% to 99% in the training cohort and from 6% to 71% in the validation cohort. These findings suggest that applying the nomogram in clinical practice could assist in identifying PAHSCI patients who may benefit from early intervention.

## 4 Discussion

The incidence of SCIWORA in pediatric spinal cord injury ranges from 19% to 34%, with PAHSCI representing its most prevalent subtype (26, 27). In recent years, the incidence of PAHSCI has risen steadily, now accounting for over 50% of SCIWORA cases (3–5). Clinically, PAHSCI is characterized by motor, sensory, and autonomic dysfunction, yet radiological examinations typically reveal no evidence of fracture or dislocation (6). Notably, a subset of PAHSCI cases is concurrently diagnosed as ATM (14). However, to date, no studies have systematically compared the distinguishing features of typical versus ATM-type PAHSCI. Accordingly, there is a pressing need for a tool to predict the likelihood of ATM complications in children with PAHSCI. Nomograms, as intuitive and quantitatively robust graphical models, are well-suited for individualized risk assessment and can inform targeted strategies for disease prevention and management. In our study, LASSO regression identified age, fall, latent activity, flow void, pinprick sensation score, and spinal cord atrophy as significant factors associated with ATM risk. The LASSO method effectively mitigated multicollinearity among candidate variables (28). Subsequently, five statistically significant variables—age, fall, latent activity, flow void, and pinprick sensation score—were identified through univariate and multivariate logistic regression and incorporated into the predictive model, which was then visualized as a nomogram. The resulting nomogram demonstrated robust discriminative performance, with AUCs of 0.876 (95% CI: 0.803–0.950) and 0.844 (95% CI: 0.709–0.979) in the training and validation cohorts, respectively. Calibration curves and decision curve analyses further supported its clinical applicability.

To date, several predictive models have been developed to aid in the differential diagnosis of transverse myelitis or to assess the prognosis of SCIWORA, with the aim of informing clinical decision-making and guiding therapeutic strategies. For example, Barreras et al. (29) constructed a prediction model using multivariate logistic regression to differentiate transverse myelitis from other causes of myelopathy, analyzing data from 457 patients. Their model achieved a multinomial AUC of 0.76 and a correct classification rate of 87%, ultimately revealing that 46% of initially diagnosed transverse myelitis cases were in fact non-inflammatory lesions (e.g., vascular malformations, degenerative compression), thereby helping to avoid unnecessary immunotherapy (29). Zhou et al. (10) developed a prognostic model for unfavorable outcomes in patients with idiopathic acute transverse myelitis based on three risk factors: absence of second-line therapies, high EDSS score at nadir, and a positive MRI result. However, the model lacked internal validation. Similarly, Wang et al. constructed a prognostic model to predict postoperative recovery in patients with cervical spinal cord injury without radiographic abnormality, incorporating disease duration, preoperative JOA score, and cervical Pavlov ratio. While the model demonstrated good internal performance (AUC = 0.894), it was not externally validated (30). To our knowledge, our study is the first to develop a predictive model specifically for PAHSCI patients with concomitant ATM, offering several notable advantages. First, the nomogram incorporates only five easily obtainable clinical variables to accurately predict the risk of ATM in PAHSCI, substantially reducing the time required for diagnostic assessment. Second, our predictive model demonstrated excellent accuracy and stability in internal validation, with AUCs of 0.876 and 0.844, respectively. Decision curve analyses further confirmed its favorable clinical utility. Finally, the nomogram provides an intuitive estimate of individual risk: clinicians can simply locate the score for each variable, sum the total, and determine the predicted probability, thereby facilitating more informed treatment decisions.

In China, PAHSCI often occurred during children’s dance waist training (16), and the proportion of spinal cord injury caused by dancing waist down in sports injuries is as high as 96.2% (31). When performing dance and other movements such as waist down, repeated hyperextension of the spine might cause spinal root vein injury, resulting in spinal vein drainage disorders, secondary ischemia due to spinal venous hypertension, and then neurological function injury (8, 32, 33). In addition, the pressure of the inferior vena cava by the abdominal organs during hyperextension of the spine and the increased pressure of the chest and abdominal cavity during the Valsalva exercise could aggravate the obstruction of spinal venous drainage (8, 34). It was suggested that when the spine was over-deformed, the spinal cord and its nutrient vessels could be injured due to longitudinal pull (6, 35). Ren’s study proposed a similar view and supported the mechanism of injury by spinal cord diffusion tensor tractography examination of two PAHSCI children showing disruption of spinal nerve fiber bundles (36). In this study, we performed LASSO regression analysis on the hyperextension-related activity patterns of children with PAHSCI. The results revealed that patients exhibiting latent activity characteristics were less likely to develop ATM-type PAHSCI but more prone to typical-type PAHSCI. These findings suggest that while hyperextension activity may not be a primary trigger for ATM comorbidity, it plays a significant role in the pathogenesis of PAHSCI itself. This may be attributable to ATM being predominantly inflammation-driven, whereas the typical form is primarily mediated by mechanical injury.

Falls are defined as unintentional events in which the body loses balance and comes into contact with the ground or a lower surface (37). Previous studies have established falls as a direct cause of spinal cord injury, typically resulting from high-energy trauma to the spine and mechanical compression of the spinal cord during the event (38). In contrast, ATM is primarily triggered by infectious or immune-mediated mechanisms and lacks a direct causal relationship with mechanical trauma such as falls. The nomogram developed in this study revealed that PAHSCI cases following a fall induced by hyperextension are more likely to be of the typical subtype and less likely to represent the ATM subtype. This observation suggests that ATM-type PAHSCI is likely driven by underlying autoimmune or infectious etiologies, with hyperextension serving as a potential precipitating factor, whereas falls appear unrelated to its pathogenesis.

Children’s vertebrae have special anatomical features, such as small joints with more flexible horizontal translation, vertebrae with anterior wedge, more flexible ligaments and joint capsules (27), which make children’s vertebrae less stable and more mobile, and more prone to spinal slide under mild external forces, resulting in spinal cord injury (39). In this study, children with the common type of PAHSCI were older than those with the ATM subtype, and increasing age was negatively associated with the likelihood of an ATM-type diagnosis. This may reflect the greater incidence of spinal cord injuries from hyperextension activities such as dancing or backbending in older children, contributing to a rise in PAHSCI cases (40). In addition, younger children, with less developed immune systems, may be more susceptible to myelitis and spinal cord injuries triggered by infections or immune-related mechanisms (41).

In many PAHSCI imaging examinations, MRI shows obvious superiority (42), and the main imaging features include spinal edema, spinal hemorrhage, spinal atrophy, and non-neurological soft tissue injury (43, 44). Many studies have pointed out that MRI can better predict the prognosis of children with PAHSCI (2, 45). Generally speaking, patients with normal MRI have better prognosis than those with abnormal MRI (36), and patients with bleeding manifestations are more severe and have poor prognosis (46, 47). ATM patients may also have MRI-positive indications, including long T2 signal in the spinal cord, spinal cord enlargement, gadolinium enhancement, etc. Besides, MRI-negative patients have a better prognosis than MRI-positive patients (10, 11). Previous studies have primarily attributed the presence of vascular flow voids in children with PAHSCI to elevated spinal venous pressure. High-intensity hyperextension may damage spinal vasculature, impair venous return, and ultimately lead to venous hypertension (8, 9). Traditionally, spinal venous hypertension has been considered a radiological marker suggestive of spinal dural arteriovenous fistula or intracranial hypotension. Our findings indicate that flow voids are more frequently observed on MRI in typical-type PAHSCI cases than in ATM-type cases. We hypothesize that in typical PAHSCI, vigorous hyperextension induces vascular injury and venous hypertension, which may serve as a primary mechanism underlying spinal cord injury. In contrast, ATM-type PAHSCI is likely driven by spinal inflammation secondary to infection or autoimmune response (11, 12). While minor hyperextension may act as a secondary trigger, it is insufficient to cause venous outflow obstruction, explaining the lower prevalence of venous hypertension features in this subgroup.

Wang et al. (8) reported that MRA in children with acute hyperextension spinal cord injury revealed tortuous, dilated dorsal spinal veins, suggesting that repeated spinal hyperextension may predispose to venous injury. Anatomically, pinprick sensation is mediated by the lateral spinothalamic tract, a region with relatively sparse vascular supply, rendering it more vulnerable to injury during hyperextension movements in PAHSCI (48). In this study, the nomogram identified pinprick sensation score as a potential predictor of ATM secondary to PAHSCI, with lower scores associated with higher ATM risk. However, its coefficient in the LASSO regression was near zero, suggesting minimal clinical relevance. Given that ATM is primarily inflammation-driven, this finding may indicate a potential pathogenic mechanism in ATM-type PAHSCI, whereby regions traversed by the spinothalamic tract, with their relatively sparse vascular supply, are more susceptible to inflammatory involvement.

## 5 Limitation

Despite these strengths, the study has certain limitations. First, the flow void may be an artifact of the CSF flow void shadow. It is suggested to adopt better strategies to reduce artifact generation, including reducing time of flight and free water elimination effects and increasing the number of excitation (45). Furthermore, in regions suspected of flow voids, it is recommended to compare findings on T2WI and FLAIR sequences; concordant low signals on both suggest cerebrospinal fluid cavities (49). Since MRA is the gold standard for diagnosing ATM, it is recommended to perform MRA for definitive diagnosis. Second, as a retrospective study, cases with incomplete data were excluded via listwise deletion—particularly as cases lacked clinical scores and neurological assessments, a process that may have introduced selection bias. Variables requiring examinations (e.g., pinprick sensation score) were more likely missing in emergency settings. Consequently, the model’s applicability may be confined to centers with dedicated spinal rehabilitation teams capable of comprehensive assessments, rather than acute care hospitals where initial triage decisions are made. Third, developed from single-centre data and internally validated via 1,000 bootstrap resamples, the model lacks external validation in independent cohorts and is thus not yet generalizable to other populations. As a preliminary construct, its clinical applicability requires confirmation through multicentre prospective studies. Fourth, our study exhibited a gender imbalance, with females constituting 83% of all participants (Table 1). This may be because dance training accounts for 96.2% of sports-related injuries in China. This may limit the generalizability of the model. We propose its use as a specialized classification tool for managing dance-induced PAHSCI in girls within rehabilitation centers. Before broader application, external validation in more diverse populations is required.

## 6 Conclusion

This study expands the clinical understanding of PAHSCI and enhances recognition of its diagnosis and classification. Through a systematic retrospective analysis of 130 cases from our institution, we identified 20 risk factors associated with ATM in children with PAHSCI. Using LASSO regression and multivariate logistic regression, we distilled these to five key predictors and developed a preliminary nomogram to assist clinicians in assessing the risk of concurrent ATM. This tool has the potential to inform clinical decision-making and help avoid unnecessary antimicrobial or immunomodulatory treatments.

## Data Availability

The raw data supporting the conclusions of this article will be made available by the authors, without undue reservation.
