# Supplementary material for: Development and validation of a predictive model for acute myelitis secondary to hyperextension-induced spinal cord injury in pediatric patients
Source: Front Neurol. 2025 Oct 24;16:1629920. doi: 10.3389/fneur.2025.1629920 (PMC12604103; doi:10.3389/fneur.2025.1629920)
Supplement: Supplementary file 1 [file Table_1.DOCX]

| **Predictor** | **β-coefficient** | **Direction of Effect** |
| --- | --- | --- |
| **(intercept)** | 1.275611e+00 | - |
| **fall** | -5.286376e-01 | Significant |
| **latent activity** | -4.830252e-01 | Significant |
| **flow void** | -4.049422e-01 | Significant |
| **age** | -1.544616e-01 | Significant |
| **pinprick sensation score** | -1.448668e-04 | Marginal |
| **spinal cord atrophy** | 7.499332e-17 | Excluded (LASSO compressed) |

**Supplementary Datasheet 1.** Non-zero coefficient variables and their corresponding coefficients.

Optimal λ= 0.092 (selected via 10-fold cross-validation)
